# Factors Associated with Sexual Activity for Women with Pelvic Floor Dysfunction - A Cross-Sectional Study

**DOI:** 10.1055/s-0040-1713805

**Published:** 2020-09-08

**Authors:** Sandra Rebouças Macêdo, José Ananias Vasconcelos Neto, José Tadeu Nunes Tamanini, Leonardo Bezerra, Rodrigo Aquino Castro

**Affiliations:** 1 Department of the Physiotherapy Course, Centro Universitário Unichristus, Fortaleza, CE, Brazil; 2 Department of Urogynecology, Universidade Federal de São Carlos, São Carlos, SP, Brazil; 3 Department of Medicine, Hospital Geral de Fortaleza, Fortaleza, CE, Brazil; 4 Department of Maternal and Child Health, Universidade Federal do Ceará, Fortaleza, CE, Brazil; 5 Department of Gynecology, Escola Paulista de Medicina, Universidade Federal de São Paulo, São Paulo, SP, Brazil

**Keywords:** sexual activity, pelvic floor dysfunction, urinary incontinence, pelvic organ prolapsed, atividade sexual, disfunções do assoalho pélvico, incontinência urinária, prolapso do assoalho pélvico, qualidade de vida, quality of life

## Abstract

**Objective** To examine women with pelvic floor dysfunction (PFDs) and identify factors associated with sexual activity (SA) status that impacts quality of life (QoL).

**Methods** We conducted a cross-sectional study that included women > 18 years old who presented with at least one PFD symptom (urinary incontinence [UI] and/or pelvic organ prolapse [POP]), in outpatient clinics specializing in urogynecology and PFD in Fortaleza, state of Ceará, Brazil, using a service evaluation form and QoL questionnaires.

**Results** The analysis of 659 women with PFD included 286 SA (43.4%) women and 373 non-sexually active (NSA) (56.6%) women, with a mean age of 54.7 (±12) years old. The results revealed that age (odds ratio [OR] = 1.07, 95% confidence interval [CI] 1.03–1.12) and post-menopausal status (OR = 2.28, 95% CI 1.08–4.8) were negatively associated with SA. Being married (OR = 0.43, 95% CI 0.21–0.88) was associated with SA. Pelvic organ prolapse (OR = 1.16, 95% CI 0.81–1.68) and UI (OR = 0.17, 95% CI 0.08–0.36) did not prevent SA. SF-36 Health Survey results indicated that only the domain functional capacity was significantly worse in NSA women ( *p* = 0.012). Two King's Health Questionnaire domains in NSA women, impact of UI ( *p* = 0.005) and personal relationships ( *p* < 0.001), were significantly associated factors. Data from the Prolapse Quality-of-life Questionnaire indicated that NSA women exhibited compromised QoL.

**Conclusion** Postmenopausal status and age negatively affected SA. Being married facilitated SA. Presence of POP and UI did not affect SA. However, NSA women with POP exhibited compromised QoL.

## Resumo

**Objetivo** Examinar mulheres com disfunções do assoalho pélvico (DAP) e identificar fatores associados ao status de atividade sexual (AS) e impacto na qualidade de vida (QV).

**Métodos** Realizamos um estudo transversal, no qual participaram mulheres > 18 anos, que apresentaram pelo menos um sintoma de DAP (incontinência urinária [UI] e/ou prolapso de órgão pélvico [POP]), em ambulatórios especializados em uroginecologia e DAP em Fortaleza, CE, Brasil, utilizando um formulário de avaliação de serviço e questionários de QV.

**Resultados** A análise de 659 mulheres com DAP incluiu 286 mulheres sexualmente ativas (SA) (43,4%) e 373 mulheres não sexualmente ativas (NSA) (56,6%), com idade média de 54,7 (±12) anos. Os resultados revelaram que idade (odds ratio [OR] = 1,07; intervalo de confiança [IC] 95%: 1,03–1,12) e status pós-menopausa (OR = 2,28; IC 95% 1,08–4,8) foram negativamente associados à atividade sexual. O casamento (OR = 0,43; IC 95% 0,21–0,88) foi associado à AS. Por outro lado, POP (OR = 1,16; IC 95% 0,81–1,68) e IU (OR = 0,17; IC 95% 0,08–0,36) não impediram a AS. Os resultados do SF-36 Health Survey indicaram que apenas a capacidade funcional do domínio ( *p* = 0,012) foi significativamente pior em mulheres NSA. Dois domínios King's Health Questionnaire (KHQ, na sigla em inglês) em mulheres NSA, impacto da IU ( *p* = 0,005) e relacionamento pessoal ( *p* < 0,001), foram fatores significativamente associados. Os dados do Prolapse Quality-of-life Questionnaire (P-QoL, na sigla em inglês) indicaram que as mulheres NSA apresentavam QV comprometida.

**Conclusão** O status pós-menopausa e a idade afetaram negativamente a AS, enquanto o casamento facilitou a AS. A presença de POP e IU não afetou a AS. No entanto, as mulheres NSA com POP apresentaram QV comprometida.

## Introduction

In the context of clinical practice, women who are sexually active (SA) and those who are non-sexually active (NSA) with satisfactory self-perception of their sexual function (SF) may also experience sexual problems related to pelvic floor dysfunction (PFD). 1

The reported prevalence of sexual activity in middle-aged and older women ranges from 53 to 79%, depending on the population studied. 2 Panman et al 3 reported that, among older women with PFD, increased age and lower levels of education were predictors of sexual inactivity.

Sexual activity and SF rates have not been found to exhibit any differences between women with and without PFD, and women with PFD are as likely to be SA as those without PFD. 4 5 However, women with pelvic organ prolapse (POP) are reported to be more likely to avoid sexual activity compared with women with urinary incontinence (UI). 4 5 Previous studies have reported that PFD strongly affects perimenopausal and postmenopausal women. The demand for evaluation and treatment of these conditions has steadily increased in recent years, with increased life expectancy and efforts to promote quality of life (QoL). 4 5 Sexuality is a fundamental part of human life, and is an important parameter for health and QoL. 6 Thus, the current study sought to identify factors that affect sexual activity in women with PFD, and the impacts of QoL, according to sexual activity status.

## Methods

### Study Design and Inclusion/Exclusion Criteria

We conducted a cross-sectional observational study, which was designed for outpatient clinics specializing in urogynecology and PFD in Fortaleza, state of Ceará, Brazil. The present study was approved by the Ethics and Research Committee of the Universidade Federal de São Paulo (UNIFESP, in the Portuguese acronym), of the Hospital Geral Cesar Cals and of the Hospital Geral de Fortaleza, through the Brazil Platform (CAAE 15961313.6.0000.5505). Patients who participated in the study signed the Free Informed Consent Terms prepared according to Resolution 466/2012 of the National Health Council. In the present study, 659 women with PFD were examined between September 2013 and November 2016. The eligibility criteria included women > 18 years old who presented with at least one symptom of PFD (UI and/or POP). The exclusion criteria included severe cognitive impairment, psychiatric and neurological diseases and pelvic cancer.

### Independent Variables and Dependent Variables

Data were collected during outpatient follow-up of participants using a standardized form collecting sociodemographic data, clinical features and physical examinations. We administered questionnaires that have been translated into Portuguese and validated, including the QoL general questionnaire SF36 Health Survey (SF-36), 7 as well as condition-specific questionnaires such as King's Health Questionnaire (KHQ) 8 and the Prolapse Quality-of-life Questionnaire (P-QoL). 9

Medical evaluation was performed using a standardized anamnesis and physical gynecological examination standardized with Pelvic Organ Prolapse Quantification (POP-Q) proposed by the International Continence Society (ICS), classifying the point of greatest prolapse with reference to the hymenal caruncle in stages from zero to four. 10 In relation to POP, women were classified as POP stage < II or stage ≥ II.

The diagnosis of UI type was based on clinical complaints, according to the standardization developed by the ICS. The condition was then classified according to the International Consultation on Incontinence Questionnaire - Short Form (ICIQ-SF) total score, as slight (1–5), moderate (6–12), severe (13–18) or very severe (19–21). 11 12

Perineal sensitivity and anal reflex were tested to investigate the integrity of the motor component of the pudendal nerve: bulbocavernosus cough and anocutaneous reflexes. Evaluation of the muscular function of the pelvic floor muscles (PFMs) was performed using the Perfect-Oxford Scheme (modified), on a scale of 0 to 5 13 and muscle strength. Power (P) and endurance (E) were analyzed as components.

Fecal incontinence and chronic pelvic pain were not analyzed in the current study, as the number of cases was too small to have sufficient statistical power.

### Statistical Analysis

For the variables, the data were presented as means and standard deviations (SDs), or medians, with 25th and 75th percentiles. For categorical variables, the participants were exposed to frequency to investigate associations between factors related to sexual activity.

In the analysis of the characteristics of the groups, the Mann-Whitney U-test was used because the data did not adhere to the Gaussian distribution, indicating non-normality in the sample distribution. A significance level of 5% was adopted. The Pearson chi-squared test and the Fisher exact test were used for categorical variables to investigate the associations between the variables. Logistic regression analysis was used to verify the factors influencing sexual activity status.

Variables that exhibited *p* < 0.20 in the univariate model were included in the multiple logistic regression model. Statistical analyses were performed using IBM SPSS Statistics for Windows, Version 22.0 (IBM Corp., Armonk, NY, USA) and the R 3.3.1 (The R Foundation, Vienna, Austria) software.

## Results

The results of the present study were based on an analysis of 659 women with PFD, categorized into 2 main groups: 286 SA women (43.4%) and 373 NSA women (56.6%) with PFD.

To describe the clinical diagnoses of PFD of the women and examine the relationships with sexual activity, the variables were divided into UI and POP. Urinary incontinence was classified as mild, moderate, severe and very severe according to the ICIQ-SF final score classification. Pelvic organ prolapse was classified according to the POP-Q system as stage < II or stage ≥ II.

The results were organized into blocks of data from women with SA and NSA status with PFD regarding: a) clinical and demographic characteristics ( Table 1 ); b) characteristics according to the type of PFD ( Table 2 ); c) characteristics according to the physical examination ( Table 3 ); d) logistic regression analyses related to the association between clinical, demographic and PFD characteristics ( Table 4 ); and e) to QoL scores ( Table 5 ).

**Table 1:** Distribution of clinical and demographic characteristics of women with pelvic floor disorder according to sexual activity

Demographic and clinical characteristics of patients	Sexually active	Non-sexually active	*p-value*
Age (Md ± SD) ( *n* = 642)	47 ± 9	61 ± 12	< 0.00 aa
Marital status, n (%) ( *n* = 627)			< 0.00 bb
Single	37 (38.1)	60 (61.9)	
Married	215 (57.5)	159 (42.5)	
Divorced	22 (35.5)	40 (64.5)	
Widowed	9 (9.6)	85 (90.4)	
Education b (Md ± SD) ( *n* = 608)	7 ± 4	6 ± 4	< 0.00 aa
Family income (Md ± SD) ( *n* = 519)	1.243 ± 812	1.293 ± 1365	0. 045 aa
Number of deliveries (Md ± SD) ( *n* = 647)	3 ± 2	5 ± 4 ^a^	< 0.001 aa
Diabetes, n (%) ( *n* = 659)			0.967 aa
No	280 (43.4)	365 (56.5)	
Yes	6 (42.9)	8 (57.1)	
SAH, n (%) ( *n* = 659)			0.021 aa
No	257 (45.2)	312 (54.8)	
Yes	29 (32.2)	61 (67.8)	
Obesity, n (%) ( *n* = 659)			0.004 aa
No	251 (41.7)	351 (58.3)	
Yes	35 (61.4)	22 (38.6)	
BMI (Md ± SD) ( *n* = 491)	29.5 ± 4.9	28.3 ± 5.1 ^c^	0.007 bb
Menopause, n (%) ( *n* = 564)			< 0.001 aa
No	100 (66.1)	82 (33,9)	
Yes	82 (23.4)	268 (76.5)	
Do not know (Hysterectomized)	17 (53.1)	15 (46.9)	
Smoking, n (%) ( *n* = 636)			0.003 aa
Never smoked	174 (46.9)	197 (53.1)	
Previously smoked	67 (32.5)	139 (67.5)	
Currently smoking	28 (47.5)	31 (52.5)	

Abbreviations: BMI, Body mass index; Md ± SD, mean and standard deviation; SAH, systemic arterial hypertension.

aa Mann-Whitney U test.

b Years of study.

bb Pearson's Chi-square test.

**Table 2:** Distribution of characteristics according to the type of pelvic floor disorder and sexual activity status

PFD	Sexually active	Non-sexually active	*p-value*
UI ( *n* = 521)	239 (45.9)	282 (54.1)	< 0.001 aa
POP stage, n (%) ( *n* = 640)			< 0.001 aa
POP stage <II	248 (50.7)	241 (49.3)	
POP stage ≥II	28 (18.5)	123 (81.5)	
TVL, (Md ± SD) ( *n* = 634)	10 ± 1	9 ± 2	< 0.001 bb
GH (Md ± SD) ( *n* = 635)	4 ± 1	4 ± 2	0.678 bb
PB (Md ± SD) ( *n* = 636)	4 ± 1	3 ± 1	< 0.001 bb

Abbreviations: GH, genital hiatus; Md ± SD; mean and standard deviation; POP, pelvic organ prolapse; PB, perineal body; TVL, total vaginal length; UI, urinary incontinence.

aa Pearson's Chi-square test.

bb Mann-Whitney U test.

**Table 3:** Distribution of characteristics according to the physical examination of women with pelvic floor disorder according to sexual activity

Physical exam	Sexually active	Non-sexually active	*p-value*
Perineal sensitivity, n (%) ( *n* = 555)			0.568 a
No	10 (50)	10 (50)	
Yes	231 (43.2)	304 (56.8)	
Anal reflex, n (%) ( *n* = 534)			0.247 a
No	42 (41.2)	60 (58.8)	
Yes	187 (43.3)	245 (56.7)	
Muscle strength * (Md ± SD) ( *n* = 368)	1.9 ± 1.3	1.6 ± 1.3	0.079 b
Endurance # (Md ± SD) ( *n* = 368)	3.4 ± 2.8	2.9 ± 2.7	0.097 b

Abbreviations: Md ± SD; mean and standard deviation.

a Pearson's Chi-square test.

b Mann-Whitney U test.

* Oxford scale.

# Sustained contraction time in seconds.

**Table 4:** Results of the logistic regression analyses of the association of clinical, demographic and type of pelvic floor dysfunction according to sexual activity

Variables	Univariate logistic regression OR (CI 95%)	p-value	Multivariate logistic regression * OR (CI 95%)	p-value
Age (Md ± SD) ( *n* = 642)	1.12 (1.09–1.14)	< 0.001	1.07 (1.03–1.12)	< 0.001
Marital status, n (%) ( *n* = 627)				
Single f	–	–	–	–
Married	0.45 (0.28–0.72)	< 0.001	0.43 (0.21–0.88)	0.021
Divorced	1.12 (0.57–2.17)	0.735	1.03 (0.39–2.74)	0.941
Widowed	5.82 (2.61–12.96)	< 0.001	2.66 (0.69–10.3)	0.155
Education d (Md ± SD) ( *n* = 608)	0.94 (0.91–0.98)	0.003	1.05 (0.98–1.13)	0.116
Number of deliveries ( *n* = 647)	1.17 (1.10–1.25)	< 0.001	1.06 (0.94–1.19)	0.342
Menopause ( *n* = 564)				
Yes e	6.32 (4.40–9.08)	< 0.001	2.28 (1.08–4.8)	0.030
Obesity ( *n* = 659)	0.44 (0.25–0.78)	0.005	1.39 (9.54–3.58)	0.489
Smoking ( *n* = 636) Never smoked f	–	–	–	–
Previously smoked	1.83 (1.28–2.61)	< 0.001	1.06 (0.59–1.93)	0.826
Currently smoking	0.97 (0.56–1.69)	0.936		
BMI ( *n* = 491)	0.95 (0.92–0.98)	0.010	0.97 (0.91–1.03)	0.411
TVL ( *n* = 634)	0.74 (0.66–0.82)	< 0.001	0.81 (0.66–0.99)	0.041
PB( *n* = 636)	0.75 (0.65–0.86)	< 0.001	1.01 (0.78–1.31)	0.912
UI ( *n* = 521)	0.17 (0.08–0.36)	< 0.001	0.000	0.999
POP stage, n (%) ( *n* = 640)				
POP stage <II	1.19 (0.84–1.69)	0.322	0.55 (0.17–1.75)	0.316

Abbreviations: BMI, body mass index; CI, confidence interval; Md ± SD; mean and standard deviation; OR, odds ratio; POP, pelvic organ prolapse; PB, perineal body; TVL, total vaginal length; UI, urinary incontinence.

d Years of study.

e Versus no menopause.

f Variable reference used for variables with more than two response options.

* Multivariable logistic regression model.

**Table 5:** Distribution of quality of life questionnaire scores of women with pelvic floor disorder according to sexual activity

Quality of life questionnaire	Sexually active	Non-sexually active	*p-value* ^a^
SF 36 Med (25th–75th percentile) ( *n* = 494)			
GHP score	52 (32–72)	57 (35–72)	0.254
Functional capacity	60 (35–85)	50 (25–75)	0.012 *
Daily activities limitations	25 (0–100)	0 (0–100)	0.495
Emotional limitations	33 (0–100)	33 (0–100)	0.559
Social limitations	75 (50–100)	75 (37.5–100)	0.811
Vitality	50 (30–70)	52.5 (30–80)	0.467
Pain	51 (31–62)	51 (32–72)	0.234
Mental health	60 (44–80)	64 (44–80)	0.371
KHQ Med (25th–75th percentile) ( *n* = 568)			
GHP score	50 (25–75)	75 (25–75)	0.028
UI impact	66.6 (33.3–100)	100 (66.6–100)	0.005 *
Daily activities limitations	50 (16.6–66.6)	50 (16.6–83.3)	0.345
Physical limitations	50 (16.6–83.3)	50 (16.6–100)	0.174
Social limitations	22.2 (0–44.4)	33.3 (0–55.5)	0.100
Personal relationships	33.3 (0–66.6)	0 (0–33.3)	< 0.001 *
Emotions	44.4 (22.2–77.7)	49.9 (22.2–88.8)	0.334
Sleep and disposition	33.3 (16.6–66.6)	50 (0–83.3)	0.370
Measure of gravity	53.3 (33.3–73.3)	46.6 (33.3–73.3)	0.733
PQol Med (25th–75th percentile) ( *n* = 640)			
GHP score	50 (25–75)	50 (50–75)	0.202
Impact prolapsed	33.3 (33.3–100)	100 (33.3–100)	< 0.001 *
Impact on daily activities	16.6 (0–66)	50 (0–100)	< 0.001 *
Physical limitations	16.6 (0–50)	33.3 (0–83.3)	0.003 *
Social limitations	0 (0–22.2)	11.1 (0–44.4)	< 0.001 *
Personal relationships	33.3 (0–50)	0 (0–50)	0.009 *
Emotions	33.3 (11.1–66.6)	44.4 (22.2–88.8)	0.203
Sleep/energy	16.6 (0–50)	16.6 (0–50)	0.490
Severity	25 (8.3–37.4)	33.3 (16.6–50)	0.003 *
Total PISQ 12 score	28 (22–34)	–	–

Abbreviations: GHP, general health practice; KHQ, King's health questionnaire; Med (25th–75th percentile), median and percentile (25–75%); PQol, prolapse quality of life; PISQ, Pelvic organ prolapse/urinary incontinence sexual questionnaire; SFQ, short form health survey; UI, urinary incontinence.

* *p* < 0.20.

The mean age of women in the present study was 54.7 (±12.6) years old. Women in the NSA group were older on average than those in the SA group ( *p* < 0.001). Most women in both groups were married. Sexually active women were younger and had a higher level of education, evaluated by the duration of studying. The education level of SA women (7 ± 4 years) was higher than that of NSA women (6 ± 4 years) ( *p* < 0.001).

Table 1 lists the clinical and demographic characteristics of women with PFD, according to their sexual activity status. The results revealed that age ( *p* < 0.001), marital status ( *p* < 0.001), education ( *p* < 0.001), number of births ( *p* < 0.001), obesity ( *p* = 0.004), body mass index (BMI) ( *p* = 0.007), menopause ( *p* < 0.001) and smoking ( *p* < 0.001) were significantly different between the SA and NSA groups, while family income, diabetes ( *p* = 0.967) and systemic arterial hypertension (SAH) ( *p* = 0.021) were not significantly different between groups.

Being married (odds ratio [OR] = 0.43) appeared to have a protective effect on maintaining sexual activity. Exhibiting menopause (OR = 2.28) and being widowed (OR = 2.66) affected the ability to maintain sexual activity. In addition, the results revealed that these variables have relevant confidence intervals (CIs) - menopause (1.08–4.8), widowed (0.39–2.74). In the present study, the main reasons reported for sexual inactivity were the lack of a partner or the presence of disease (cardiovascular or neurological) in a partner.

The groups showed significant differences in demographic characteristics according to the type of PFD ( Table 2 ). In relation to the POP stage ( *p* < 0.001), POP < II, was predominant. However, SA women were more likely to exhibit POP < II while NSA women were more likely to exhibit POP ≥ II. Women with UI were predominantly in the NSA group ( *n* ***=*** 282) ( *p* < 0.001).

Total vaginal length (TVL) ( *p* < 0.001) and perineal body (PB) ( *p* < 0.001) were significant factors when compared among the groups. Sexually active women exhibited a higher mean TVL than NSA women. The mean TVL was 10 (±1) in the SA group and 9 (±2 cm) in the NSA group ( *p* < 0.001).

Regarding physical factors ( Table 3 ), the following variables were not significant: perineal sensitivity ( *p* = 0.568), anal reflex ( *p* = 0.247), muscle strength ( *p* = 0.079) and endurance ( *p* = 0.097),

In the descriptive analysis of the present study, several variables were identified that exhibited significant differences between SA and NSA: age ( *p* < 0.001), marital status ( *p* < 0.001), education ( *p* < 0.001), number of births ( *p* < 0.001), obesity ( *p* = 0.004), BMI ( *p* = 0.007), menopause ( *p* < 0.001), smoking ( *p* = 0.003), UI ( *p* < 0.001), POP ( *p* < 0.001), TVL ( *p* < 0.001) and PB ( *p* < 0.001).

We performed a univariate logistic analysis ( Table 4 ) to identify variables related to sexual activity, revealing a range of factors with significant associations, including age ( *p* < 0.001), marital status (married; [ *p* = 0.021], divorced [ *p* = 0.941], widowed [ *p* = 0.155]), education ( *p* = 0.116), number of deliveries ( *p* = 0.342), menopause ( *p* = 0.030), obesity ( *p* = 0.489), current smoking status (previously smoked; *p* = 0.826), TVL ( *p* = 0.041), PB ( *p* = 0.912), and UI ( *p* = 0.999).

However, when these variables were adjusted using multivariate logistic regression ( Table 4 ), age ( *p* < 0.001), menopause ( *p* = 0.030) and TVL ( *p* = 0.041) were significantly associated with the absence of sexual activity. However, the analysis of TVL between groups revealed a mean difference of 1 cm between the SA and NSA groups. Considering that this is a consequence rather than a cause of maintaining sexual activity, we decided not to include this finding in the results of the present study.

The results revealed that older patients were more likely to be NSA. In addition, being married was shown to have a protective effect against being NSA. Compared with single women, widows were 2.6 times more likely to be NSA. Women at menopause were twice as likely to be NSA. Women with very severe UI were three times more likely to be NSA compared with women with mild UI.

The results of the QoL questionnaires (SF-36, KHQ and PQoL) ( Table 5 ) revealed that the groups did not exhibit significant differences in General Perceptual Health (GPH) evaluation variables. The PQoL contained a larger number of domains exhibiting significant associations.

## Discussion

The multivariate logistic regression results in the present study highlighted age and menopause as important factors associated with NSA status among women with PFD.

Panman et al 3 also identified age as a predictor of sexual inactivity, in addition to low levels of education. Thomas et al, 2 in a large cross-sectional study, reported that a large proportion of middle-aged and older women (61.2%) expressed that they would remain SA if they had a partner.

Özengin et al 14 conducted a retrospective study with 721 women, concluding that POP type did not affect SF, muscle strength, colorectal symptoms or urinary symptoms. The need for further studies examining the causes of sexual inactivity in women, as well as the effect of the presence of a partnership, was highlighted by the authors. 14 Importantly, in the present study, we verified that 82 (23.4%) SA women and 268 (76.5%) NSA women were postmenopausal. The hormonal status variable was significantly related to sexual activity ( *p* < 0.001). It has been estimated that, by 2050, 33% of women in the United States will have postmenopausal status. 15 Ringa et al 16 analyzed various components of sexuality and reported that women did not differ in sexual activity according to hormonal status. The results suggested that the hormonal and biological changes that characterize menopause do not negatively affect sexual activity. 16 Among women with PFD in the current study, 286 were SA (43.4%) while 373 were NSA (56.6%). A study by Fashokun et al 4 reported that women with PFD were as likely to be SA as women without PFD, reporting that sexual activity and SF rates did not differ between women with and without PFD.

In the current study, the POP classification was divided into stage < II or stage ≥ II. In our sample, in most women (in both the SA and NSA groups) with POP, there was an association with UI. Regarding the POP stage, POP < II was most common. However, SA women mostly exhibited POP < II, while NSA women mostly exhibited POP stage ≥ II.

Espuña-Pons et al 17 reported that the only independently-associated variables were age, symptom of vaginal protuberance, and difficulty having intercourse due to the sensation of protuberance.

Rogers highlighted the importance of providing women with reassurance and evidence that POP does not prevent sexual activity. 18

Comparison of SA and NSA women in the present study revealed that NSA women exhibited greater severity of POP, with 123 (81.5%) exhibiting POP stage ≥ II. Jha et al 5 reported that the absence of sexual activity among women with POP was greater than that among women with UI. Moroni et al 19 claimed that, among women with POP, the anatomical features of the prolapse did not appear to interfere with genital body image or with sexual function. In addition, the presence of POP was not found to be associated with being sexually active or inactive. 19

In the current study, we found UI in 239 (45%) and 282 (54.1%) women in the SA and NSA groups, respectively. Similar rates of UI in women have been reported in other studies, which also indicated that age, marital status and UI-independent predictors of sexual inactivity, negatively affected female sexual activity status, in addition to UI. 20

The general perceptual health variables of the QoL instruments used (SF-36, KHQ and PQoL) in SA and NSA women in the present study did not exhibit significant differences between groups. In contrast, a previous study reported that QoL decreases with age and decreasing hormonal levels. 21

In the present study, the SF-36, which only revealed a significant result for the functional capacity variable ( *p* = 0.012), was used to determine the QoL of women with PFD according to sexual activity.

The questionnaires can provide a useful complement to patient history and self-reported results, and were developed to assess the symptoms, the degree of incompatibility and QoL in patients with PFD. 22

In the present study, only two KHQ domains exhibited relevant results: impact of UI and personal relationships. A study by Karbage et al 22 indicated that UI affects sexual activity and QoL.

The results of the median scores of the questionnaires among women with PFD in the present study revealed significant effects for a range of PQoL variables: impact of POP ( *p* < 0.001), impact on daily activities ( *p* < 0.001), physical limitations ( *p* = 0.003), social limitations ( *p* < 0.001), personal relationships ( *p* = 0.009) and severity ( *p* = 0.003).

It is relatively common for QoL to be compromised in POP. In accordance with the current findings, a study by Svihrova et al 23 using the PQoL reported that women with POP exhibited severely impaired QoL.

In the study population in the present study, the analysis of socioeconomic characteristics of SA and NSA women with PFD revealed that the mean age was 47 (±9) years old in SA women, and 61 (±12) years old in NSA women. Thus, many older women were SA, while PFD was negatively associated with sexual activity. As reported by Fashokun et al, 4 patient age significantly differed between women with and without PFD.

Most women in the present study, in both groups, were married or in stable relationships. The average household income was higher among SA women. In the period of the survey, the average income was equivalent to approximately two minimum wages. Similar results regarding marital status and income were reported by Martins et al. 24

In the current study, TVL was found to be significantly associated with the likelihood of SA (10 ± 1) ( *p* < 0.041). Based on the current findings, and on those of other studies reporting that TVL is not relevant to sexual activity, we did not include these data in our study, as we believe that such a finding is likely to be a consequence rather than a causal factor in maintaining sexual activity. 25

Since the present study had a cross-sectional design, it was not possible to determine causality. However, despite this limitation, the current findings demonstrated an association of UI and POP with the maintenance of sexual activity. A further limitation of the present study was an inability to identify the causes of sexual inactivity among women. In addition, although some women reported that the lack of a partner or the presence of a disease (cardiovascular and neurological) was the cause of sexual inactivity, this issue requires more comprehensive investigation.

## Conclusion

The current results revealed that age and postmenopausal status negatively affected sexual activity, while being married facilitated sexual activity in women with PFD. Presence of POP and UI did not affect sexual activity. Non-sexually active women with genital prolapse exhibited significantly impaired QoL compared with SA women.
